# Grain growth of NpO_2_ and UO_2_ nanocrystals†

**DOI:** 10.1039/d3ra00487b

**Published:** 2023-02-22

**Authors:** Viktoria Baumann, Karin Popa, Marco Cologna, Murielle Rivenet, Olaf Walter

**Affiliations:** a Univ. Lille, CNRS, Centrale Lille, Univ. Artois, UMR 8181 – UCCS – Unité de Catalyse et Chimie du Solide F-59000 Lille France viktoria.baumann@centralelille.fr; b European Commission, Joint Research Centre Karlsruhe Germany olaf.walter@ec.europa.eu

## Abstract

We report on the crystallite growth of nanometric NpO_2_ and UO_2_ powders. The AnO_2_ nanoparticles (An = U and Np) were synthesized by hydrothermal decomposition of the corresponding actinide(iv) oxalates. NpO_2_ powder was isothermally annealed between 950 °C and 1150 °C and UO_2_ between 650 °C and 1000 °C. The crystallite growth was then followed by high-temperature X-ray diffraction (HT-XRD). The activation energies for the growth of crystallites of UO_2_ and NpO_2_ were determined to be 264(26) kJ mol^−1^ and 442(32) kJ mol^−1^, respectively, with a growth exponent *n* = 4. The value of the exponent *n* and the low activation energy suggest that the crystalline growth is rate-controlled by the mobility of the pores, which migrate by atomic diffusion along the pore surfaces. We could thus estimate the cation self-diffusion coefficient along the surface in UO_2_, NpO_2_ and PuO_2_. While data for surface diffusion coefficients for NpO_2_ and PuO_2_ are lacking in the literature, the comparison with literature data for UO_2_ supports further the hypothesis of a surface diffusion controlled growth mechanism.

## Abstract 2

We report on the crystallite growth of nanometric NpO_2_ and UO_2_ powders.

## Graphical abstract

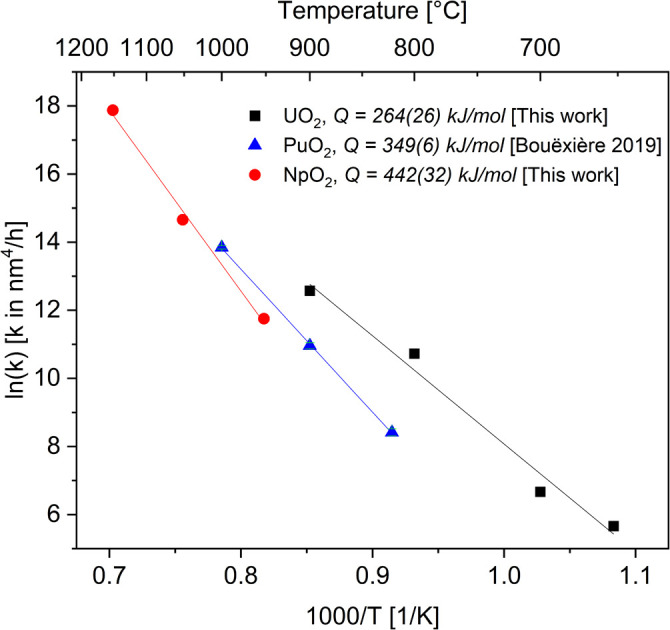

## 1 Introduction

In the processing of ceramic materials sintering is one of the most important and energy-intensive steps. Nuclear fuel pellets (UO_2_ or (U,Pu)O_2_) are made by cold pressing the powder in a die and then heating to high temperatures until the particles have coalesced. To sinter such powders to the required relative density of 95%, temperatures up to 1700 °C are typically used.^1–3^

The densification rate is a function of particle size and temperature. If the particle size is decreased, the densification rate will increase, and the temperature and duration of the process can be decreased, saving costs and energy.

In the last decade, different syntheses routes of nanocrystalline AnO_2_ have been presented.^5–10^ The hydrothermal decomposition of actinide(iv) oxalates to nanocrystalline actinide dioxide powder has shown several advantages^11–14^ and the potential to decrease drastically the sintering temperature.^15,16^

In order to control the sintering process and the final microstructure, its associated mechanisms must be well understood. Sintering is accompanied by grain growth and the elimination of pores. Such processes can occur through different mechanisms as surface diffusion, grain boundary diffusion, lattice diffusion or vapor transport.^4,17^ In crystalline ceramics, grain boundary (GB) and lattice diffusion (L) from grain boundary to pore contribute most to the densification stage, while diffusion from the surface (S) leads to neck-growth but not to densification. The activation energies *Q* for the diffusion coefficients are typically in the order *Q*_L_ > *Q*_GB_ > *Q*_S_, and surface diffusion is thus dominating at low temperature, which is one of the reasons why fast-firing techniques employ a rapid heating rate in the low temperatures, to overcome the non-densifying range.^18–20^

The migration of pores in nuclear fuels is also controlled by a surface diffusion or evaporation–condensation mechanisms.^21^*In situ*-methods can provide valuable information on the mechanisms involved in sintering, grain growth and pore-migration. Environmental scanning electron microscope at high temperature (HT-ESEM) was introduced as an innovative and powerful method to investigate *in situ* the sintering behaviour of actinide oxide materials.^22–25^ For example, Clavier *et al.* used HT-ESEM device to obtain sintering maps of ThO_2_, as well as an impressive 2-grain scale observation to capture the first stage of sintering.^26^ Bouëxière *et al.* measured the crystallite growth of PuO_2_ nanocrystals *in situ* with a HT-XRD device, obtaining an activation energy for crystallite growth of 351(5) kJ mol^−1^.^27^ In this work we applied the same method to UO_2_ and NpO_2_ nanocrystals. The samples were isothermally annealed for 30 hours in the range of 950 °C to 1150 °C for NpO_2_, and 650 °C to 1000 °C for UO_2_. During this process, XRD patterns were recorded so that particle growth could be determined over time at the respective temperatures. We calculated activation energies for UO_2_ and NpO_2_ and elaborated on the crystallite growth exponent.

## 2 Experimental

### 2.1 Synthesis

**Fig. 1:**
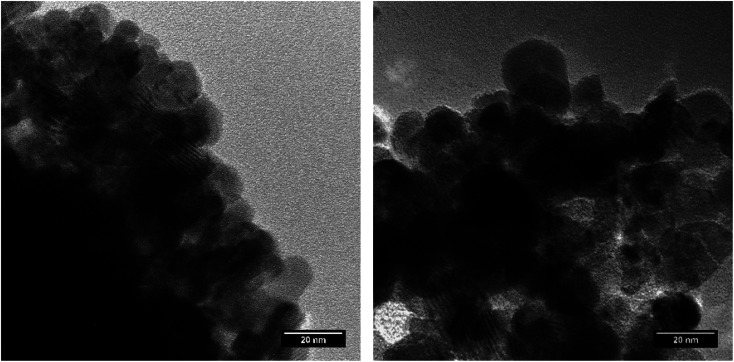
TEM images of NpO_2_ (left) and UO_2_ (right) obtained by hydrothermal decomposition of the corresponding actinide oxalates.

Nanocrystalline NpO_2_ and UO_2_ powders, shown in Fig. 1, were prepared by the hydrothermal decomposition of the corresponding actinide oxalates into actinide dioxide nanoparticles, as described elsewhere.^12^ The process was reported for the first time by Walter *et al.*^11^ and has already been applied in several studies.^12,15,28^ In summary, the oxalates (U(C_2_O_4_)_2_·6H_2_O and Np(C_2_O_4_)_2_·6H_2_O), were directly precipitated from a U(iv) solution (0.47 M, obtained by electroreduction of a UO_2_(NO_3_)_2_ solution in 4 M HNO_3_ with 0.5 M hydrazine) and from a Np(iv) solution (0.6 M in about 2 M HNO_3_) with excess of 0.5 M oxalic acid. The oxalic acid dihydrate was supplied by Merck in analytical grade. The respective oxalates were placed in a Teflon-lined hydrothermal synthesis reactor and covered with 3 to 5 ml of distilled water. The reactor was tightly sealed and heated to the desired temperature in a heating jacket made out of steel. While uranium oxalate completely decomposed after only 3.5 hours at a temperature of 170 °C, neptunium oxalate was heated at 160 °C for 18 hours. To avoid possible oxidation of UO_2_, the work was carried out under argon atmosphere.

### 2.2 High-temperature XRD

The isothermal HT-XRD measurements were performed under vacuum using a Bruker D8 diffractometer with a Bragg–Brentano configuration, a curved Ge-(1,1,1) monochromator, a ceramic copper tube (40 kV, 40 mA), and an Anton Paar HTK 2000 chamber. Approximately 10 mg of sample was mixed with 1 ml of ethanol and the suspension was placed on the Pt sample holder preheated at 70 °C to ensure homogeneous distribution of the powder during evaporation of ethanol. The chamber was closed, vacuumed and heated to the desired temperature. For the isothermal experiments, a temperature range of 650 °C to 1000 °C was chosen for UO_2_. For NpO_2_, a temperature range of 700 °C to 1150 °C was investigated and due to the faster growth, a range of 950 °C to 1150 °C was selected for the isothermal tests.

The neptunium sample was first heated at 950 °C with a heating rate of 10 °C min^−1^ and held at this temperature for 30 hours, and then annealed at 1050 °C and 1150 °C following the same procedure. 50 diffractograms were recorded at each temperature in the range of 45° < 2*Θ* < 61°, with each measurement lasting 36 minutes. A similar procedure was performed with uranium sample. However, because of the broader peak at 46.8°, the three XRD patterns were recorded in the range of 43° < 2*Θ* < 60°. In contrast to NpO_2_, fresh UO_2_ nanocrystals were used for each isothermal annealing. In this case, after reaching the desired temperature (which was maintained for 30 hours), the HT-XRD device was cooled to room temperature and the powder was then replaced with new sample before being heated to the next higher temperature. The isothermal measurement for UO_2_ was performed at 650 °C, 700 °C, 800 °C, 900 °C and 1000 °C.

### 2.3 Crystallite size measurements

The crystallite size (*G*) was calculated from the broadening of the three measured peaks. Profile fitting was performed using HighScore software (version 3.0.4) and the full width at half maximum intensity (FWHM) was calculated to determine the crystallite size using the Scherrer equation (eqn (1)):1
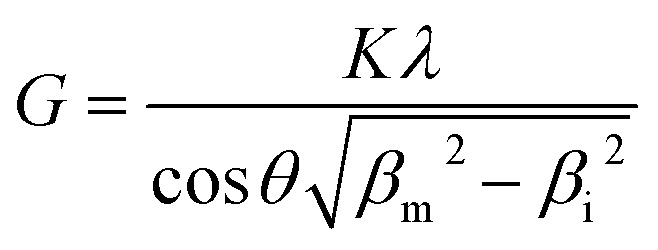
with *K* = 0.94 for spherical crystals with cubic symmetry, *β*_m_ the measured FWHM and *β*_i_ = 0.07° for the instrumental broadening. Eqn (1) was used only for *G* not larger than 150 nm, because otherwise large errors occur when *β*_m_ approaches *β*_i_. The values given for crystallite sizes are an average of the size during the 36 minute recording time of the first XRD spectrum, since some growth occurs during this time. The crystallite size of the starting oxides was 7.8(0.9) nm for UO_2_ and 7.5(1.6) nm for NpO_2_, while the lattice parameter was 5.467(2) Å and 5.441(1) Å for UO_2_ and NpO_2_, respectively. The size measured by XRD was confirmed to be consistent with the one measured by TEM in nanopowders produced in the same way.^12^

### 2.4 Grain growth model

We analyzed the grain growth kinetics of NpO_2_ and UO_2_ nanocrystallites with the classical grain growth model for porous single phase materials, as previously done for PuO_2_ nanocrystallites.^12^ The grain size data as a function of annealing time at different temperatures were fitted with (eqn (2)):^4,29^

2 *G*^*n*^ = *G*_0_^*n*^ + *kt*

where *G* is the grain size at time *t*, *G*_0_ the initial grain size, *n* the grain growth exponent, which value depends on the mechanisms of grain growth, and *k* is the grain growth rate constant, which is a function of temperature:3
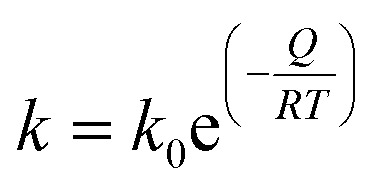
where *k*_0_ is a constant, *Q* the activation energy of the rate-controlling mechanism, *R* the gas constant, and *T* the annealing temperature. Rearranging the terms in eqn (2) and plotting *G*^*n*^ − *G*_0_^*n*^ against *t* gives a straight line with a slope equal to *k*. For each isotherm, the value of *k* was determined in this way. Performing the natural logarithm on both sides of eqn (3) yields the eqn (4):4
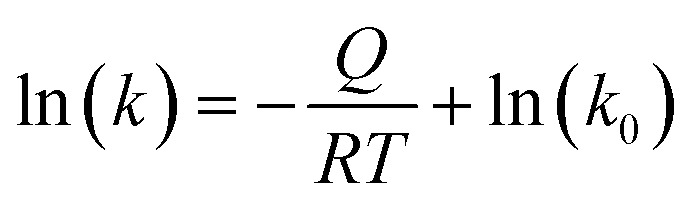


The subsequent plot of ln(*k*) as a function of 1/*T* gives the value of −*Q*/*R* as the slope of the straight line, which, multiplied by *R*, corresponds to the activation energy *Q* of the mechanism for grain growth.

## 3 Results

**Fig. 2:**
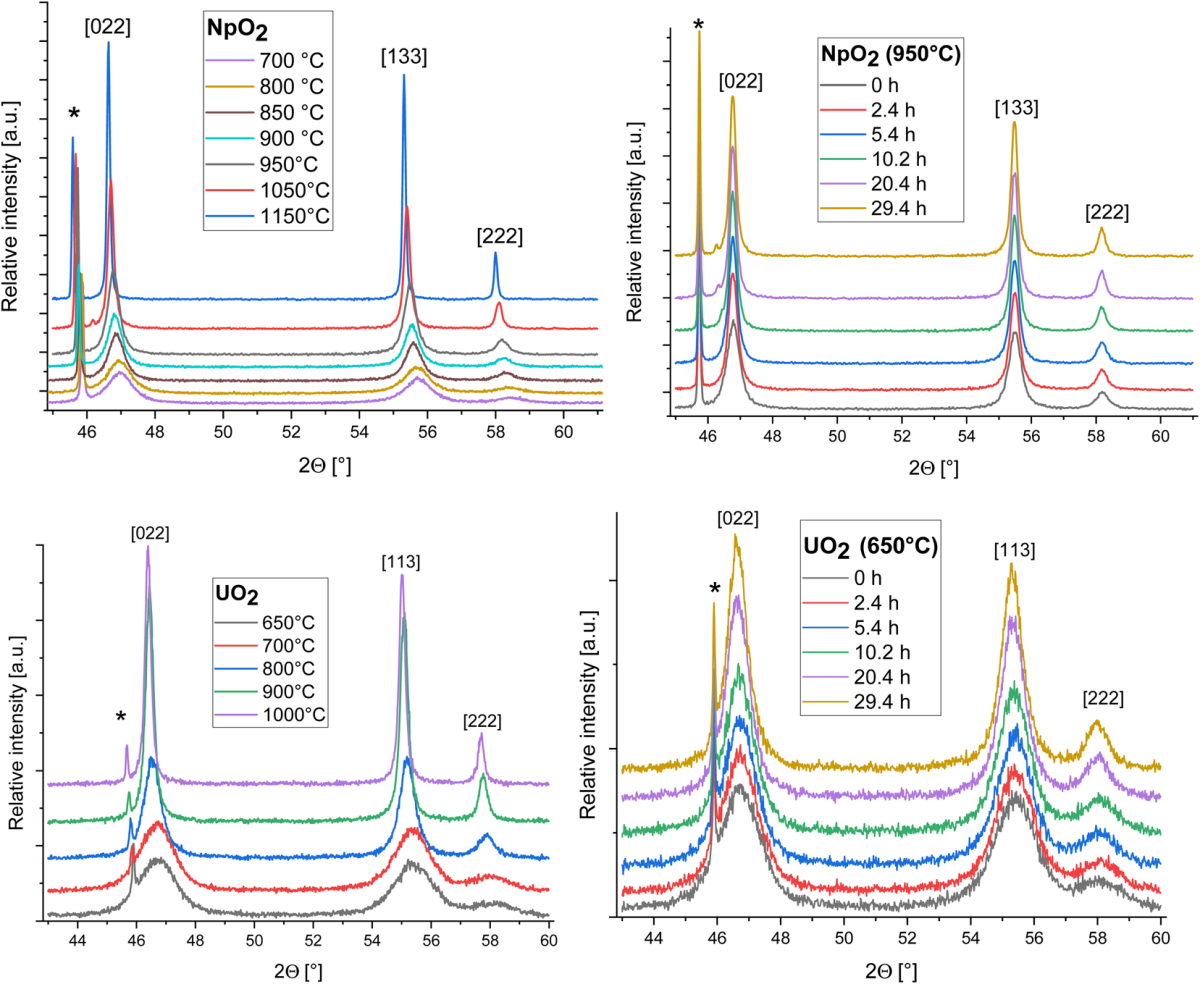
Evolution of the XRD patterns of NpO_2_ (top) and UO_2_ (down) as a function of temperature (at *t*_0_) (left) and time (at a temperature of 950 °C for NpO_2_ and 650 °C for UO_2_) (right); peaks marked by * arise from the Pt sample holder.

The XRD patterns of AnO_2_ (An = Np and U) recorded at different temperatures are shown in Fig. 2. For NpO_2_ at lower temperatures the growth is slow and *T* = 950 °C, 1050 °C and 1150 °C were chosen for the kinetic studies.

**Fig. 3:**
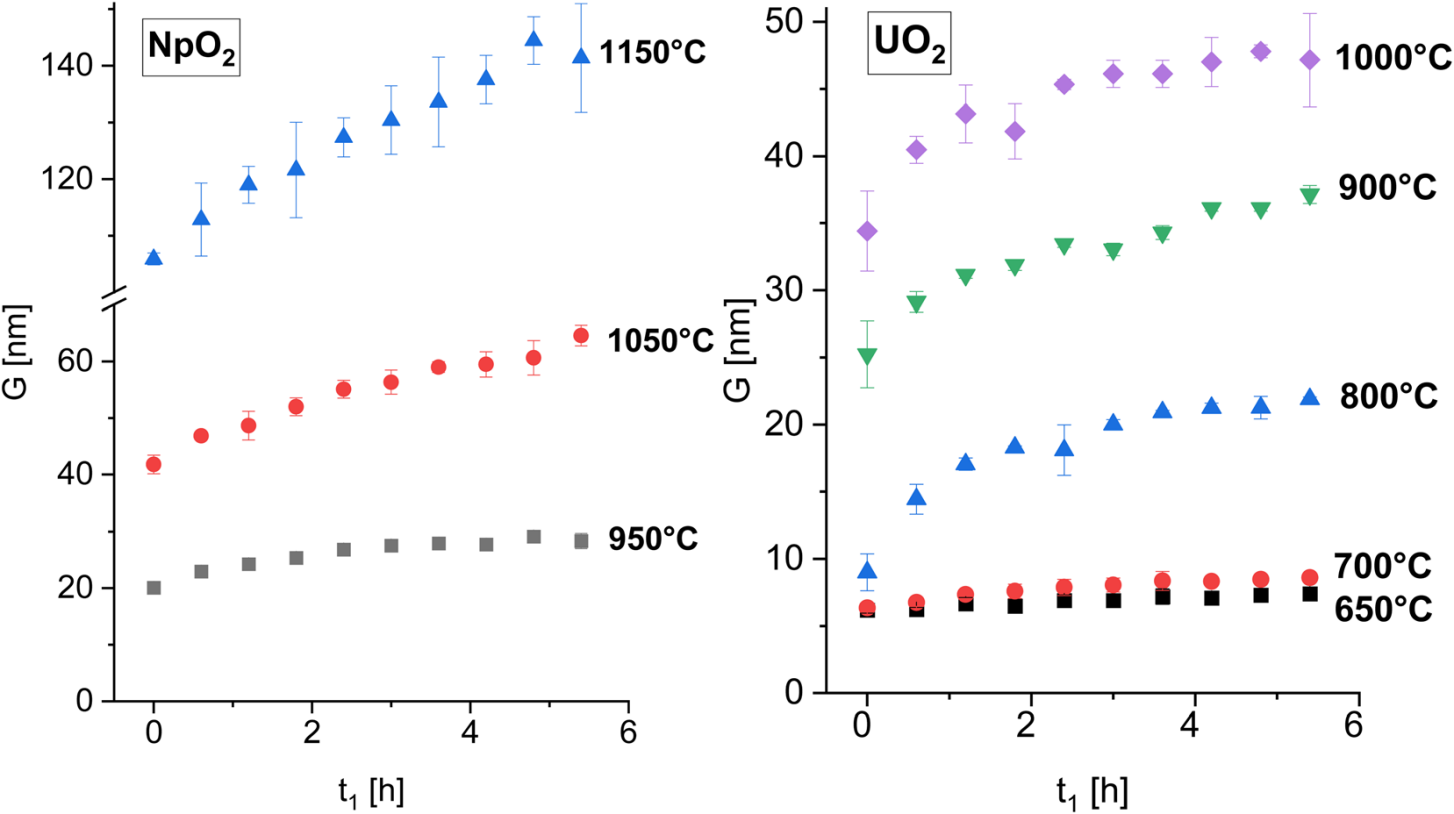
The average crystallite size of NpO_2_ (left) and UO_2_ (right) as a function of time for various temperature for the first 6 hours. The full time interval is shown in Fig. S1.†

The powder formed after hydrothermal conversion of actinide oxalates had a fluorite-type cubic structure and crystallized in the space group *Fm*3̄*m* (225). A spherical nearly shape was found for both crystallites from the transmission electron micrographs (Fig. 1). The XRD data of the isotherms measured at 950 °C (for NpO_2_) and at 650 °C (for UO_2_) are given on the right side of Fig. 2 as a function of time. The time *t*_0_ corresponds to the time after the acquisition of the first XRD spectrum (36 minutes) and consequently *t*_1_ = 72 min. The patterns of both samples showed a sharpening of the peaks with temperature and time, caused by crystallite growth. The crystallite size, as a function of time for the first 6 hours are presented in Fig. 3 and in Table 1, the full range (30 h) is shown in Fig. S1.†

**Table 1:** Calculated average crystallite size of AnO_2_ (An = U and Np) as a function of time at different temperatures. The graphical representation of these values can be seen in Fig. 3

Time [min]	NpO_2_			UO_2_				
	Crystallite size [nm]			Crystallite size [nm]				
	950 °C	1050 °C	1150 °C	650 °C	700 °C	800 °C	900 °C	1000 °C
36	20.1(0.9)	41.8(1.7)	105.9(1.1)	6.2(0.2)	6.4(0.1)	9.0(1.4)	25.2(2.5)	34.4(3.0)
72	22.9(0.5)	46.8(0.2)	112.9(6.5)	6.2(0.2)	6.8(0.3)	14.4(1.1)	29.1(0.8)	40.5(1.0)
108	24.2(0.6)	48.7(2.5)	119.0(3.3)	6.7(0.5)	7.3(0.3)	17.0(0.5)	31.1(0.2)	43.1(2.2)
144	25.4(0.6)	52.0(1.6)	121.6(8.5)	6.5(0.2)	7.6(0.5)	18.3(0.2)	31.9(0.4)	41.8(2.1)
180	26.8(0.7)	55.1(1.6)	127.4(3.5)	6.9(0.4)	7.9(0.6)	18.1(1.9)	33.4(0.3)	45.3(0.4)
216	27.5(0.3)	56.3(2.2)	130.4(6.0)	6.9(0.4)	8.1(0.5)	20.0(0.4)	33.0(0.5)	46.1(1.0)
252	27.9(0.3)	59.0(1.0)	133.6(7.9)	7.2(0.6)	8.4(0.7)	20.9(0.2)	34.3(0.5)	46.1(1.0)
288	27.7(0.9)	59.5(2.2)	137.5(4.3)	7.1(0.4)	8.3(0.4)	21.2(0.4)	36.1(0.3)	47.0(1.8)
324	29.1(0.8)	60.7(3.1)	144.4(4.2)	7.3(0.4)	8.5(0.3)	21.3(0.8)	36.1(0.3)	47.8(0.5)
360	28.3(1.3)	64.6(1.8)	141.4(9.6)	7.4(0.4)	8.6(0.3)	21.9(0.2)	37.1(0.7)	47.2(3.5)

**Fig. 4:**
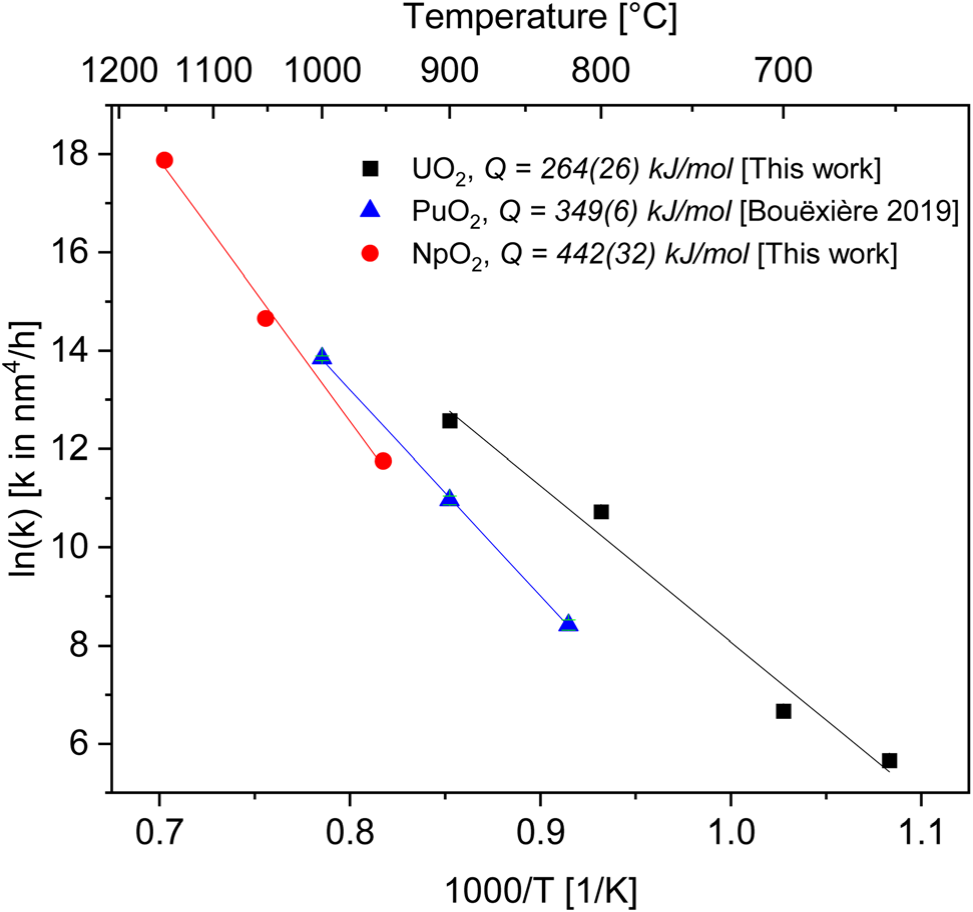
Arrhenius plot for the grain growth constant *k* using the exponent *n* = 4 for UO_2_ (black), PuO_2_ (ref. 27) (blue) and NpO_2_ (red).

After the initial rapid growth phase in the first hours, growth with slower kinetics was observed at most annealing temperatures, so that the data after 6 hours were not included in the analysis of the rate constant. Miao *et al.* also observed a similar phenomenon for the annealing of UO_2_.^30^ The grain growth exponent *n* and the growth constant *k* were determined from eqn (2) applied to the first 6 hours of annealing, by plotting *G*^*n*^ − *G*_0_^*n*^*vs.* time at constant temperature and performing a linear regression with a fixed intercept at 0. The exponent *n* was restricted in the range *n* = 2 to 4. The value of *n* = 4 was chosen because it gave the best linear fit of the experimental points as measured by the adjusted *R*^2^ values (Fig. S2 and S3†). The grain growth constant *k* is the slope of the straight line, and is listed in Table 2 together with the results of the linear fit. The activation energy was then obtained from eqn (4) as the slope of the linear fit of the natural logarithm of *k* as a function of the reciprocal temperature (1/*T*) (Fig. 4). In the case of UO_2_ we neglected the data at *T* = 1000 °C in the calculation of *Q* because the linear fit was significantly worse than those obtained for the other temperatures (Table 2), which is as well true for all other exponents *n*. The activation energies for *n* in the range *n* = 2 to 4 are given in Fig. S4† and the fitted crystallite size extrapolated to the full experimental range of 30 h in Fig. S5.†

**Table 2:** Parameters and results of the linear fitting of the data presented in Fig. S2 and S3

	Temperature [°C]	*D*_0_ [nm]	*n*	*k* [nm^4^ h^−1^]	*R* ^2^
NpO_2_	950	20.1	4	127 243(4952)	0.99
	1050	41.8	4	2 319 034(51 642)	0.99
	1150	105.9	4	57 871 100(1 661 392)	0.99
UO_2_	650	6.2	4	288(13)	0.98
	700	6.4	4	788(28)	0.99
	800	9.0	4	45 438(177)	0.99
	900	25.2	4	288 485(1130)	0.99
	1000	34.4	4	835 046(64 084)	0.94

As a result, we obtained activation energies for crystallite growth of 264(26) kJ mol^−1^ and 442(32) kJ mol^−1^ for nanocrystalline UO_2_ and NpO_2_, respectively. Fig. 4 shows also data for PuO_2_ (ref. 27) with a recalculated activation energy of 349(6) kJ mol^−1^.

## 4 Discussion

### 4.1 Grain growth exponent *n*

Grain growth in porous ceramics is commonly analysed with a simplified approach, which assumes quasi-spherical isolated pores attached at the grain boundaries.^4^ Under such assumptions, different equations can be derived for the grain growth kinetics of pure, single-phase porous systems, depending on the rate-controlling atomic diffusion mechanism and if the movement of the boundary is limited by the pore mobility (pore control), or not (boundary control). An exponent of *n* = 2 is characteristic of growth by boundary control (as in dense materials) or by pore control *via* vapour transport, *n* = 3 of pore control *via* vapour transport or lattice diffusion, while *n* = 4 is given only by pore control by surface diffusion.

Here the exponent *n* = 4 gave the best linear fit for both UO_2_ and NpO_2_ in agreement with what observed in PuO_2_,^27^ suggesting that in our conditions the growth of AnO_2_ (An = U, Np, Pu) nanopowder is controlled by the mobility of the pores, which are migrating *via* a surface diffusion mechanism. This is not surprising, as pore migration by surface diffusion is favoured at small grain size and low temperatures as the conditions investigated here, whereas pore migration by evaporation–condensation typically dominates at larger pore sizes and temperatures.^21,31^

### 4.2 Activation energy

In ceramic oxides both the cation and the anion need to be transported to allow diffusive processes (*e.g.* grain growth, sintering, creep) to occur. The slowest species along its fastest path controls the kinetics. Since in ceramic nuclear fuels the diffusion of the metal atom is orders of magnitude slower than oxygen,^32^ the activation energy *Q* in eqn (3) represents here the one for diffusion of the actinide cation (slower specie) along the pore surface (fastest path).

The calculated activation energies with *n* = 4 are 264(26) kJ mol^−1^ for U in UO_2_, 442(32) kJ mol^−1^ for Np in NpO_2_, and 349(6) kJ mol^−1^ for Pu in PuO_2_. Thus the activation energy for the growth of AnO_2_ nanocrystallites (possibly equivalent to the activation energy for cation surface diffusion) increases in the order Np < Pu < U.

The comparison of our results with the activation energy from the literature is not straightforward: data on activation energy for surface diffusion or for grain growth in PuO_2_ and NpO_2_ are scarce or lacking, while data on UO_2_ are existing, but obtained mostly at higher temperatures and very likely larger pore sizes, where different diffusion mechanisms become dominant.

The activation energy for grain growth of UO_2_ nanocrystallites controlled by pore migration found here (264 kJ mol^−1^) is in the broad range of activation energies for grain growth reported in the literature, which is very scattered in the 100–600 kJ mol^−1^ range.^21,33–36^ A direct comparison is however not sensible because of the different mechanisms taking place for grain growth at different temperature and sizes (grain boundary, volume or evaporation–condensation) and the grain growth exponent is often assumed or measured between 2 and 4. It is important to note that the choice of the exponent *n* is crucial in determining the activation energy *Q*: for example, by varying from *n* = 2 to 4 in our analysis, the associated activation energies can be twice as high (Fig. S4†). This value of 264 kJ mol^−1^ is instead to be compared with the activation energy for surface diffusion, which was determined from old experiments and more recently molecular dynamic (MD) simulations. Matzke reviewed nine experiments and proposed an activation energy for surface diffusion of 454 kJ mol^−1^ in the 1200–1700 °C range.^32^ This surprisingly high value could be a consequence of the contribution of concurrent mechanisms at such temperatures, as grain boundary, volume and evaporation–condensation. Indeed Zhou and Olander performed more sophisticated experiments by isolating the contribution of evaporation–condensation and obtained a much lower value of 300(60) kJ mol^−1^ in the 1760–2100 °C range, which is in the same range of what found here.^37^ More recent molecular dynamics simulations for diffusion of U on the surface of nanocrystals or nanopores consistently confirm a value in the 260–320 kJ mol^−1^ range.^38–40^

### 4.3 Surface diffusion coefficient

**Fig. 5:**
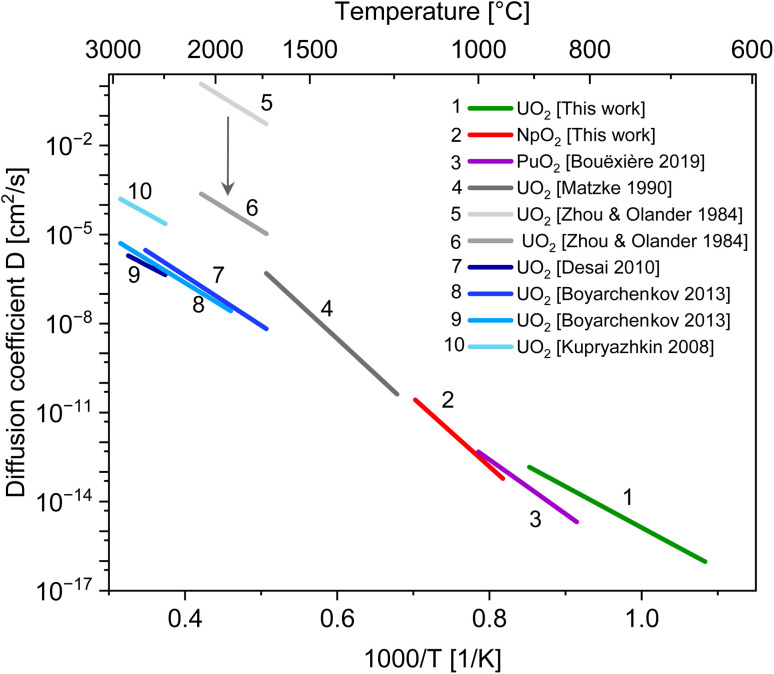
Diffusion coefficients for cation self-diffusion along the surface of actinide oxides. Values at low temperatures for U, Np and Pu are estimated from nano-grain growth experiments, lines 4 to 6 from previous experiments and lines 7 to 10 from MD simulation.

For a better comparison, we calculate the diffusion coefficient for surface diffusion *D*_s_, with the word of caution that we are performing an order-of-magnitude estimate, so the error could be in the range of 1 to 2 orders of magnitude. If, in our system, pore migration occurs by surface diffusion of U, then the grain boundary velocity *v*_b_, which can be approximated as the grain growth rate d*G*/d*t*, can be written as:^41^5
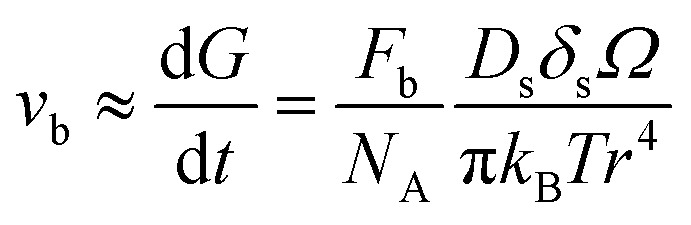
where *G* is the grain size, *F*_b_ the driving force per unit area of pore-free boundary due to its curvature, which can be expressed as *F*_b_ = *αγ*_GB_/*G*, *α* a geometrical constant having the value of 2 for spherical grains, *γ*_GB_ the grain boundary energy per unit area, taken as 1.7 J m^−2^,^42^*N*_A_ the number of pores on a unit area of the boundary ∼1/*X*^2^, with *X* the interpore distance and *X* ∼ *G*, *D*_s_ the surface diffusion coefficient, which takes the form 
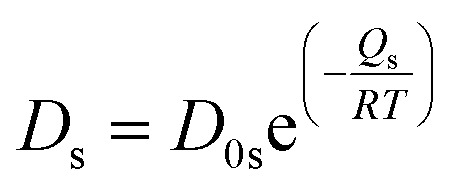
, with *D*_0s_ the diffusion pre-exponential factor and *Q*_s_ the activation energy for surface diffusion, *δ*_s_ the thickness of the surface diffusion layer (taken equal to the lattice parameter *a*, 0.54 nm),^38^*Ω* the atomic volume (4.09 × 10^−29^ m^3^), *k*_B_ the Boltzmann constant, and *r* the pore radius. Assuming coarsening by grain growth and pore coalescence (*r* ∼ *G*), eqn (5) becomes:6
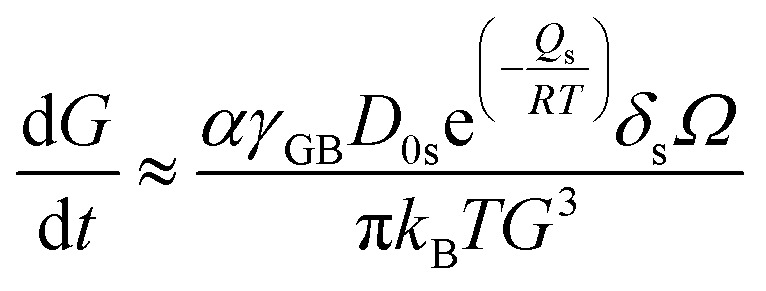
And, after integration7
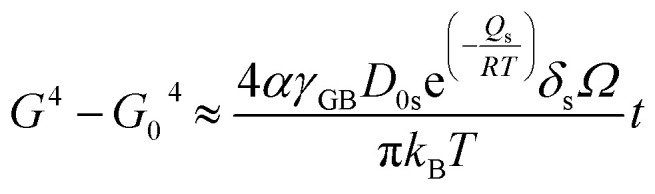
where the term to the right side is *kt* in eqn (2). Since, under the considered assumption, the activation energy in eqn (3) is *Q* = *Q*_s_, we can rearrange as:8
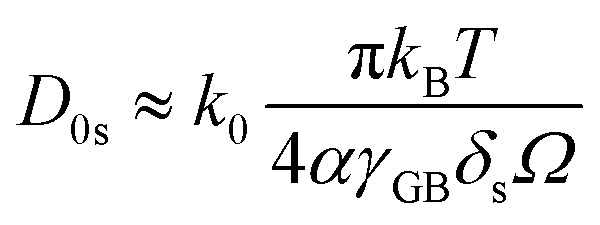
where *k*_0_ is the pre-exponential term of the grain growth constant in eqn (3) and *T* the average temperature in the considered interval. The self-diffusion coefficients *D*_s_ for U, Np and Pu, are shown in Fig. 5 and compared with literature data. The values are also summarised in Table 3.

**Table 3:** Estimated surface self-diffusion coefficient of cations in UO_2_, NpO_2_ and PuO_2_: activation energy *Q*_s_ and pre-exponential factor *D*_0s_. The latter is calculated from eqn (8), and thus we intend it as an order-of-magnitude estimate

Actinide dioxide	Temperature range (°C)	*D*_0s_ (cm^2^ s^−1^)	*Q*_s_ (kJ mol^−1^)	Method	Reference
UO_2_	650–900	8 × 10^−2^	264	Nanocrystals growth	This work
NpO_2_	950–1150	4 × 10^5^	442	Nanocrystals growth	This work
PuO_2_	820–1000	1 × 10^2^	349	Nanocrystals growth	27
UO_2_	1200–1700	5.00 × 10^5^	454	Review (species: UO_2_, UO_3_)	32
UO_2_	1760–2100	5.00 × 10^6^	301	Tracer diffusion (specie: UO_2_)	37
UO_2_	1760–2100	<10^3^	301	Tracer diffusion (specie: U^4+^)	37
UO_2_	2427–2827	4.49 × 10^−2^	257	MD simulation, nanopores	39
UO_2_	1747–2597	1.91 × 10^0^	319	MD simulation, nanocrystals	40
UO_2_	1927–2907	4.80 × 10^−1^	302	MD simulation, nanocrystals	40
UO_2_	2351–2907	4.40 × 10^0^	270	MD simulation, nanocrystals	38

The data for UO_2_ compares very well with the molecular dynamics simulations. The agreement with older experimental data is less good. One of the reason could be that competing diffusion mechanisms are involved at high temperature, as previously mentioned. The large scattering in published experimental data may also be due to the reported significant difficulties in the theoretical interpretation of tracer diffusion experiments.^32,37^ Such models were based on the assumption that the migrating species are UO_2_ and UO_3_ molecules with rotational degrees of freedom. If U^4+^ ions are assumed as the migrating species, the pre-exponential factor *D*_0_ is reduced by more than 3 orders of magnitude (see the arrow in Fig. 5).^37^

## 5 Conclusion

Activation energies of 264(26) kJ mol^−1^ and 442(32) kJ mol^−1^ were determined for UO_2_ and NpO_2_ by kinetic studies of particle growth of nanometric powder by isotherm HT-XRD measurements. For both actinide dioxides, the best linear fit was obtained with an exponent of 4, which suggests that grain growth of the nanocrystallites is controlled by the mobility of the pores (pore control), which migrate *via* a surface diffusion mechanism. Under such hypothesis, we estimated the pre-exponential term *D*_0_ of the self-diffusion coefficients *D*_s_ for U, Np and Pu in the corresponding oxides as 8 × 10^−2^ cm^2^ s^−1^ for U, 4 × 10^−2^ cm^2^ s^−1^ for Np and 1 × 10^−2^ cm^2^ s^−1^ for Pu. The satisfactory order-of-magnitude comparison of the obtained diffusion coefficient for U with literature data supports the conclusion that growth of actinides oxides nanocrystallites in the studied conditions is controlled by surface diffusion.

## Conflicts of interest

There are no conflicts to declare.

## Supplementary Material

RA-013-D3RA00487B-s001
