# Supplementary material for: Grain growth of NpO2 and UO2 nanocrystals
Source: RSC Adv. 2023 Feb 22;13(10):6414–21. doi: 10.1039/d3ra00487b (PMC9944290; doi:10.1039/d3ra00487b)
Supplement: RA-013-D3RA00487B-s001 [file RA-013-D3RA00487B-s001.pdf]

Electronic Supplementary Material (ESI) for RSC Advances.
This journal is © The Royal Society of Chemistry 2023

Supporting Information

Grain growth of NpO_2 and UO_2 nanocrystals

Viktoria Baumann[1,2,*], Karin Popa[2], Marco Cologna[2], Olaf Walter[2,**], Murielle Rivenet[1]

[1] *Univ. Lille, CNRS, Centrale Lille, Univ. Artois, UMR 8181 – UCCS – Unité de Catalyse et Chimie du Solide, F-59000 Lille, France.*
[2] *European Commission, Joint Research Centre, Karlsruhe, Germany.*
**Corresponding author. E-mail address: viktoria.baumann@univ-lille.fr; **olaf.walter@ec.europa.eu*

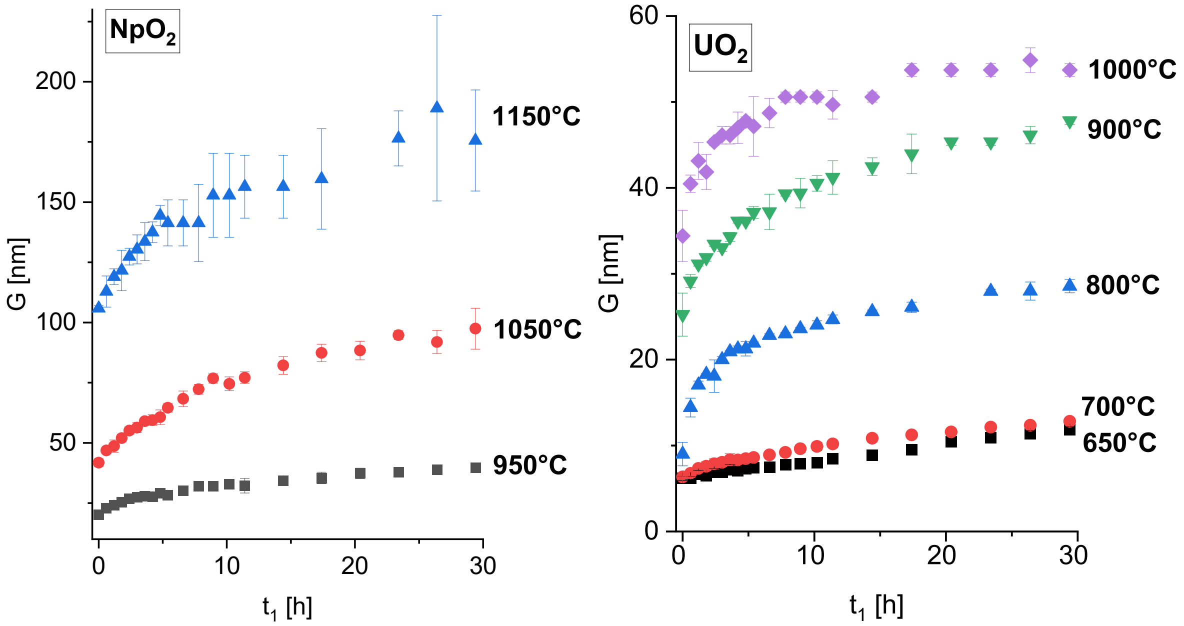

Figure S1. Crystallite size as a function of time at different temperatures for NpO_2 (left) and UO_2 (right).

The slope of the linear regression gives the grain growth constant k, reported in Table 2

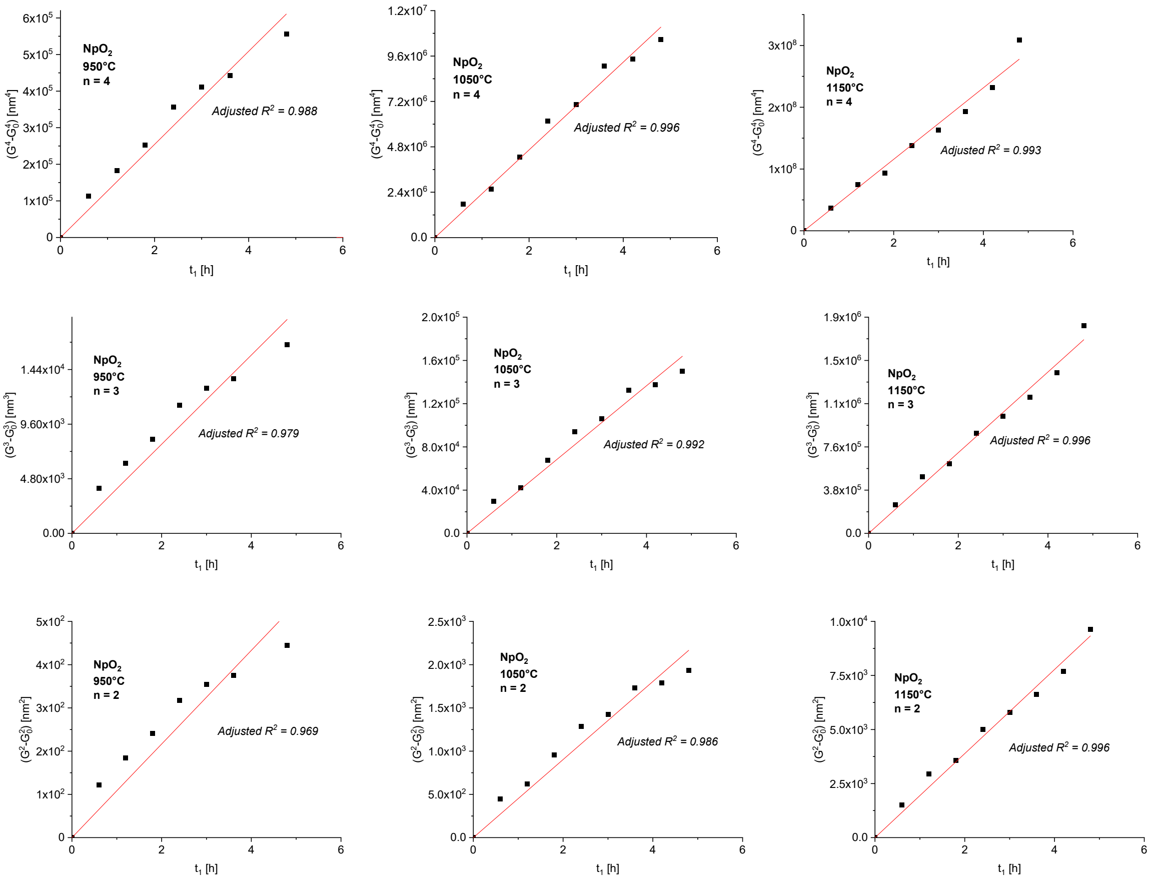

Figure S2: Crystallite growth fitted to the growth model of Eq. 2, exponent n in the range n = 2 to 4 for NpO_2

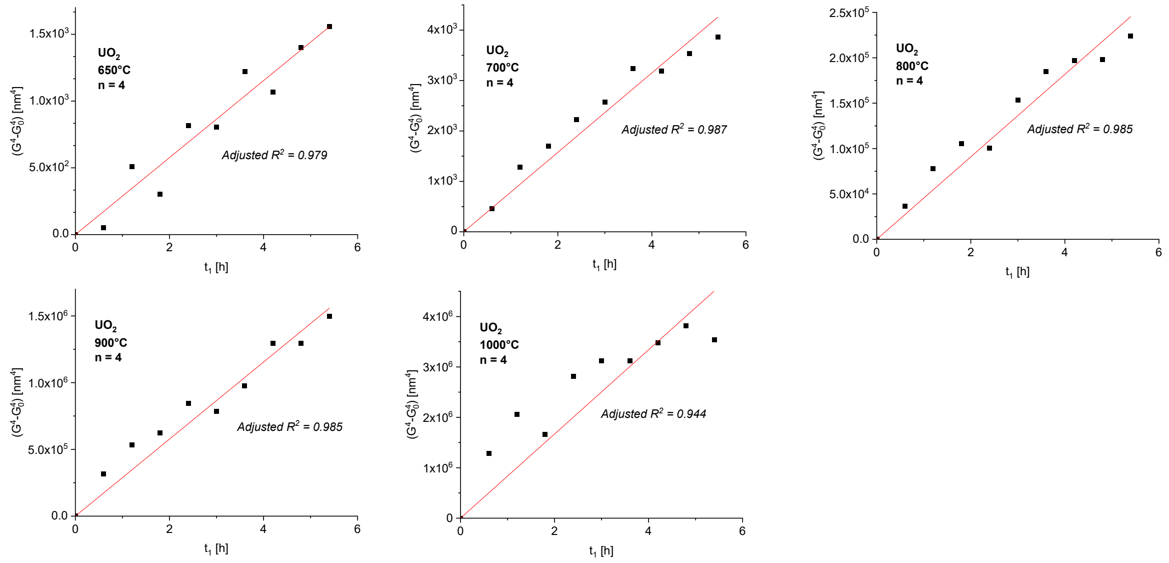

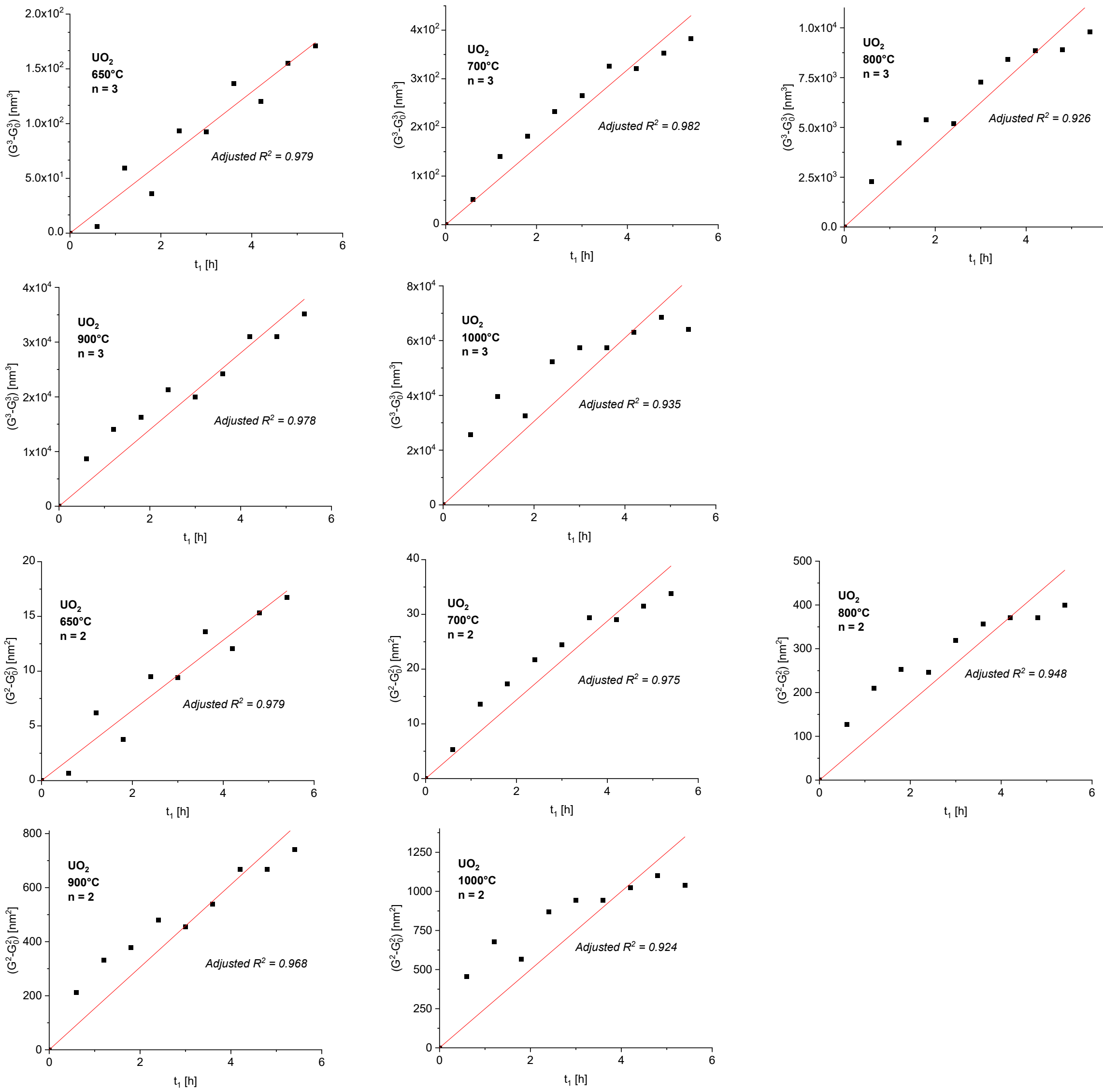

Figure S3: Crystallite growth fitted to the growth model of Eq. 2, exponent n in the range n = 2 to 4 for UO_2.

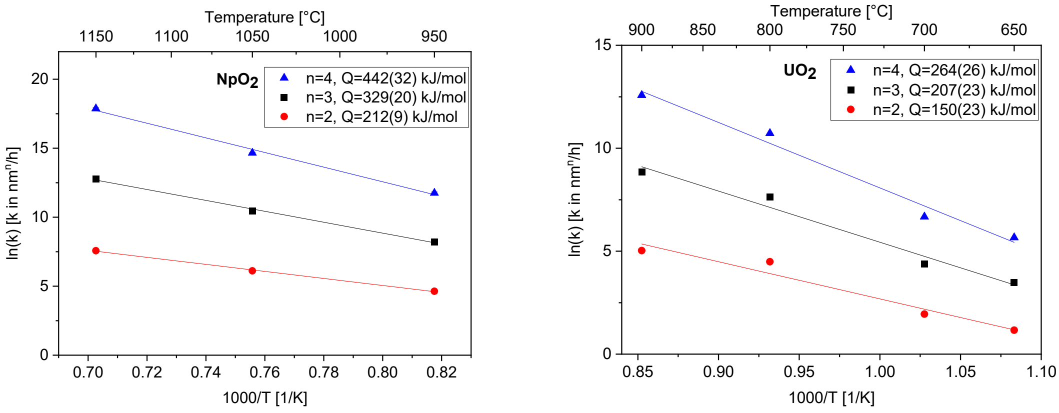

Figure S4: Arrhenius plot for exponent n in the range n = 2 to 4 for NpO_2 (left) and UO_2 (right).

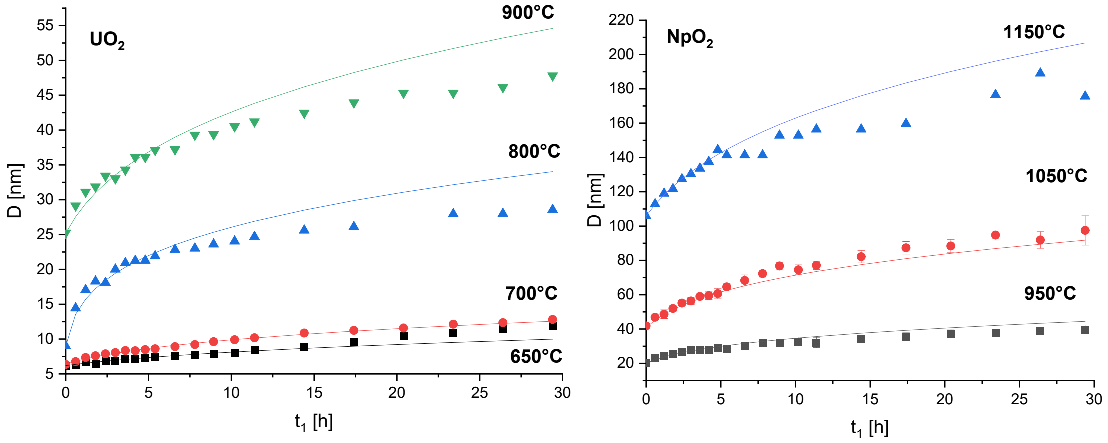

Figure S5. Extrapolation of crystallite size as a function of time.
